# Maternal sitagliptin treatment attenuates offspring glucose metabolism and intestinal proinflammatory cytokines IL-6 and TNF-α expression in male rats

**DOI:** 10.7717/peerj.10310

**Published:** 2020-11-11

**Authors:** Qian Zhang, Xinhua Xiao, Jia Zheng, Ming Li, Miao Yu, Fan Ping, Tong Wang, Xiaojing Wang

**Affiliations:** Key Laboratory of Endocrinology, Ministry of Health, Department of Endocrinology, Peking Union Medical College Hospital, Peking Union Medical College, Chinese Academy of Medical Sciences, Beijing, China

**Keywords:** Sitagliptin, Fetal programming, Inflammatory, Maternal diet, Intestine

## Abstract

Increasing evidence shows that maternal overnutrition may increase the risk of diabetes in offspring. We hypothesized that maternal sitagliptin intervention may improve glucose intolerance through gut targeting. Female Sprague-Dawley (SD) rats were fed a normal diet (ND) or a high-fat diet (HFD) for 4 weeks before mating. ND pregnant rats were divided into two subgroups: ND group (ND alone) and the ND-sitagliptin group (ND combined with 10 mg/kg/day sitagliptin treatment). HFD pregnant rats were randomized to one of two groups: HFD group (HFD alone) and the HFD-sitagliptin group (HFD combined with 10 mg/kg/day sitagliptin treatment) during pregnancy and lactation. Glucose metabolism was assessed in offspring at weaning. Intestinal gene expression levels were investigated. Maternal sitagliptin intervention moderated glucose intolerance and insulin resistance in male pups. Moreover, maternal sitagliptin treatment inhibited offspring disordered intestinal expression of proinflammatory markers, including interleukin-6 (*Il6*), *ll1b*, and tumor necrosis factor (*Tnf*), at weaning and reduced intestinal IL-6, TNF-α expression by immunohistochemical staining and serum IL-6, TNF-α levels. However, maternal sitagliptin intervention did not affect offspring serum anti-inflammatory cytokine IL-10 level. Our results are the first to show that maternal sitagliptin intervention moderated glucose metabolism in male offspring. It may be involved with moderating intestinal IL-6 and TNF-α expression in male rat offspring.

## Introduction

Currently, it is estimated that four hundred and fifteen million adults have type 2 diabetes mellitus (T2DM) worldwide (Williams et al., 2020). T2DM is characterized by abnormalities in glucose metabolism, which leads to pancreatic cell insulin secretion disorder and peripheral insulin resistance (Virally et al., 2007). T2DM and its complications create a societal and commercial burden. Genetic and environmental factors are involved in the pathology of T2DM. Traditional environmental factors mainly include lifestyle factors, including excess energy intake and sedentary lifestyle. Recent epidemiological evidence (Eriksson et al., 2014; Jornayvaz et al., 2016) and animal studies (Boucher & Leung, 2015; Ohta et al., 2017) suggest that exposure to maternal overnutrition *in utero* increases the risk of metabolic diseases, such as hypertension, cardiovascular disease, and diabetes in offspring. Researchers regard *in utero* malnutrition status as an important environmental risk factor for developing T2DM (Bianco-Miotto et al., 2017). Maternal glucose dysmetabolism needs to be reversed for the wellbeing of offspring.

Dipeptidyl peptidase (DPP)-4 inhibitors can increase the activity level and duration of action of glucagon-like peptide (GLP)-1. Thus, they have become a new class of antidiabetic drugs in recent years. Once oral nutrients are given, GLP-1 is released from intestinal L cells (Reimann et al., 2008). GLP-1 has several important blood glucose-lowering effects. However, native GLP-1 is rapidly enzymatically hydrolyzed and inactivated by DPP-4 (Kieffer, McIntosh & Pederson, 1995). Since DPP-4 inhibitors prevent the degradation of endogenous GLP-1, they have now entered the clinic to treat patients with T2DM (Lovshin & Drucker, 2009). Sitagliptin is an orally active, fully reversible DPP-4 inhibitor that was approved by the US Food and Drug Administration (FDA) in 2006 (Bergman et al., 2007). As a highly selective DPP-4 inhibitor, sitagliptin prevents degradation of GLP-1 and improves glycaemia, reduces glycated haemoglobin level, stimulates insulin secretion and suppresses glucagon secretion (Gallwitz, 2007; Pratley & Salsali, 2007). In clinical trials, sitagliptin is an effective hypoglycemic agent at every stage of type 2 diabetes (Deacon & Holst, 2013). Recently, Professor Reimer et al. administered sitagliptin to high-fat diet (HFD)-induced obese rats before pregnancy. Their results showed that this intervention strategy did not have lasting effects on fasting blood glucose in offspring (Dennison, Eslinger & Reimer, 2017). Another group reported that sitagliptin attenuates severe acute pancreatitis-associated intestinal inflammation in vivo and in vitro (Zhou et al., 2019). In a double-blind, randomized and placebo controlled clinical trial, pregnant gestational diabetes mellitus (GDM) women in the 2nd trimester were treated with sitagliptin. After 16 weeks of treatment, improved fasting blood glucose, serum insulin, HOMA-IR and HOMA-β was observed in sitagliptin treatment group (Sun et al., 2017). Another trial evaluated the combination of sitagliptin and metformin in GDM patients. Compared with metformin treatment alone, sitagliptin and metformin combination treatment had improved glucose metabolism parameters (Elkind-Hirsch et al., 2018). Sitagliptin was well-tolerated in these two trials, and did not increase the incidence of gastrointestinal side effects. To date, there has been no report on the relationship between sitagliptin and the gut in offspring that underwent *in utero* overnutrition. Furthermore, the potential intestinal mechanism remains poorly defined.

The objective of the present study was to determine the effect of maternal early sitagliptin intervention on glucose metabolism in offspring. Specifically, we examined intestinal gene expression in the offspring of dams consuming a HFD with or without sitagliptin intervention during pregnancy and lactation.

## Materials and Methods

### Animal treatments and diets

**Figure 1:**
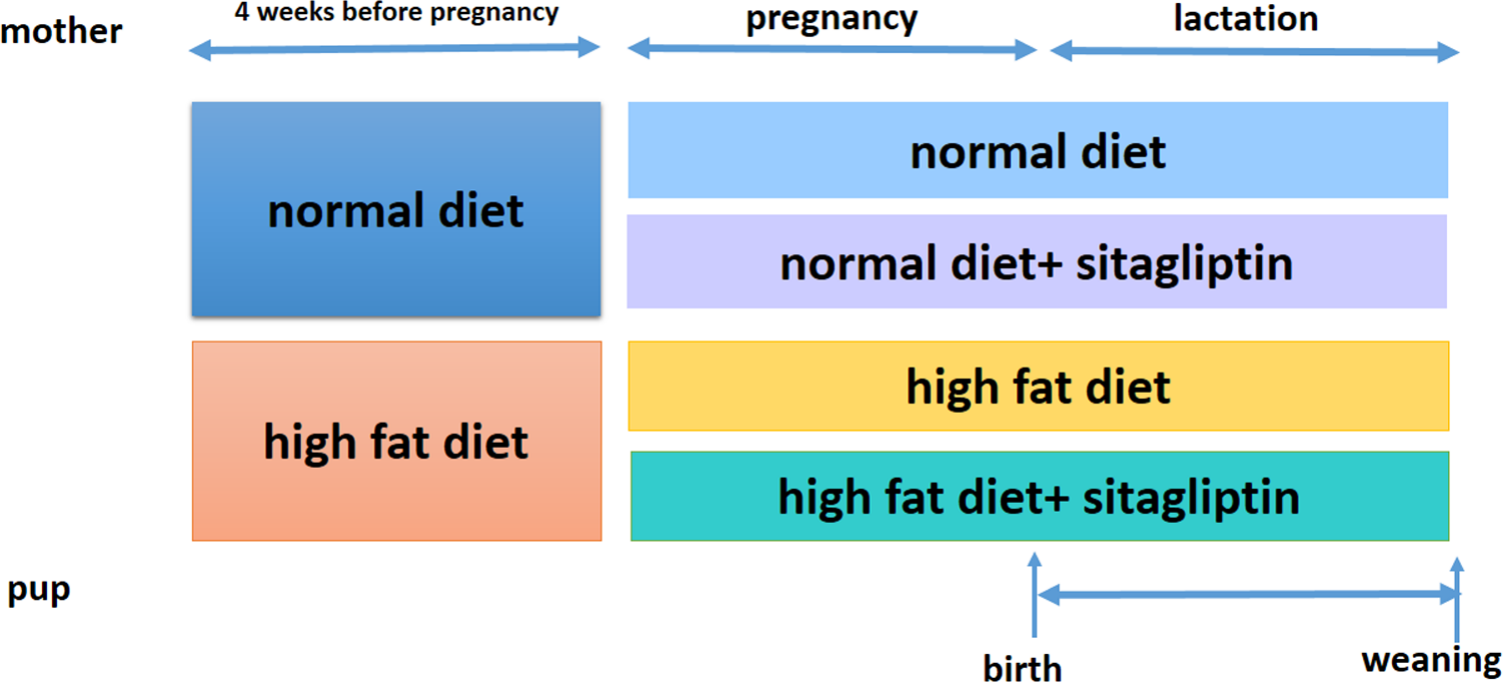
Animal experiment timeline.

Ethical approval for the study was granted by the Peking Union Medical Hospital Animal Ethics Committee (Project XHDW-2015-0051, 15 Feb 2015) and conformed to the NIH Animal Care guidelines (NIH publication No. 85-23, revised 1996). Rats were placed in a 12-h light/dark cycle and temperature (22 ± 1 °C) and humidity (65%–70%) controlled housing and were given food and water ad libitum. Eight-week-old female Sprague-Dawley rats (obtained from the Institute of Laboratory Animal Sciences of the Chinese Academy of Medical Sciences and Peking Union Medical College in Beijing, China, *n* = 32) were randomized into one of two groups for 4 weeks: normal diet (America Institute of Nutrition-93, AIN-93, kcal %: 10% fat, 20% protein, and 70% carbohydrate; 3.85 kcal/gm, ND, *n* = 16) or HFD (kcal %: 45% fat, 20% protein, and 35% carbohydrate; 4.73 kcal/gm, *n* = 16). Then, female rats were bred with male rats. After the identification of a copulation plug, dams were housed individually. To evaluate the effect of sitagliptin on mother rats with different diet and their offspring, we divided dams fed an ND into two subgroups: the ND only group and ND-sitagliptin group (*n* = 8 for each group). Meanwhile, dams fed a HFD were randomized to two subgroups: the HFD only group or the HFD-sitagliptin group (*n* = 8 for each group). The typical human daily dose of sitagliptin is 100 mg/60 kg body weight. Thus, according to the formula: d_rat_ =(37 × d_human_)/6 (Nair & Jacob, 2016), the corresponding dose of sitagliptin for rats is 10.28 mg/kg per day. Therefore, HFD-sitagliptin group rats were fed a HFD supplemented orally with 10 mg/kg/day sitagliptin treatment (Merck, West Point, PA, USA) as described in a previous study (Mega et al., 2011; Samaha, Said & Salem, 2019; Wojcicka et al., 2019). Dams treated with this protocol through pregnancy and lactation. The size of every litter was culled to 6 pups (3 male and 3 female rats) to ensure that there was no nutritional bias between litters. On the day of birth, one male offspring and one female offspring from each litter was randomly selected for experimental study (8 male and 8 female in each group). At weaning, offspring from all dams (8 male and 8 female for each group) were sacrificed following intraperitoneal injection of pentobarbital sodium (150 mg/kg). The intestines were immediately stored at −80 °C. Other surviving rats were kept feeding for other study. Fig. 1 shows the animal experimental protocol.

### Body weight, glucose tolerance, serum insulin and inflammatory cytokines assay

Dams were weighed on the day of confirmation of pregnancy and during the pregnancy. Pups were weighed on the day after birth and weaning. At weaning, pups underwent an oral glucose tolerance test (OGTT). Briefly, after overnight food deprivation, blood was sampled from the tip of the tail in rats before and 30, 60, and 120 min after oral glucose administration via gavage (2 g/kg). Blood glucose concentrations were determined by using a blood glucose meter (Contour TS glucometer, Bayer, Hamburg, Germany). The area under the glucose tolerance curve (AUC) of the OGTT was calculated as previously described (Zhang et al., 2018). At weaning, the pups were anesthetized after 10 h of fasting. Blood samples were collected from the intraorbital retrobulbar plexus. Serum insulin was measured using an ELISA kit (EZRMI-13K, Millipore, Billerica, MA, USA). Insulin sensitivity was assessed using HOMA-IR as previously described (Zhang et al., 2018). The levels of serum proinflammatory cytokines interleukin-6 (IL-6), TNF-α and anti-inflammatory cytokine IL-10 were measured using ELISA kits (RAB0311, Merck, Darmstadt, Germany, ab46070, Abcam, Cambridge, MA, USA and RAB0246 Merck, Darmstadt, Germany).

### RNA isolation, microarray processing and analysis

Previous studies showed that the programming effects of maternal high fat diet occurred in a sexually dimorphic manner (Yokomizo et al., 2014; Zheng et al., 2014), possible due to the influence of confounding factors related to female hormone profile and estrous cycle (Kleinert et al., 2018). Thus, this study mainly focused on male offspring. Total RNA was isolated from the intestines of male pups in the HFD and HFD-sitagliptin groups by using TRIzol reagent (Life Technologies Inc., Carlsbad, CA, USA). Gene expression in the intestine was detected by an Affymetrix GeneChip Rat Gene 2.0 ST whole transcript-based array (Affymetrix Technologies, Santa Clara, CA). The differential gene criteria between the two groups was 1.50-fold or higher (*p* < 0.05). The data obtained have been deposited in the NCBI Gene Expression Omnibus (GEO) database (accession number GSE134070).

The Gene Ontology (GO) classification system and Kyoto Encyclopedia of Genes and Genomes (KEGG) were used to assign biological meaning to the group of different genes and pathway enrichment through Database for Annotation, Visualization, and integrated Discovery (DAVID) software. STRING software (Biobyte Solution, Heidelberg, Germany) was used to analyze the connections among differentially expressed genes.

### Real-time PCR

To validate the gene array results, the expression of genes (*Il1b*, *Il6*, and tumor necrosis factor (*Tnf*)) was analyzed using real-time PCR. Total RNA from the four groups was reverse-transcribed by Superscript II (Life Technologies, Carlsbad, CA). The primers are shown in Table 1. Real-time PCR was performed with an ABI Prism 7500 Real-Time System (Applied Biosystems, Foster City, CA) using ABI SYBR Mix (Applied Biosystems, Foster City, CA). The mRNA levels of the target gene were corrected by glyceraldehyde 3-phosphate dehydrogenase (*Gapdh*) using the 2^−ΔΔ*Ct*^ method.

**Table 1:** Oligonucleotide sequences for qPCR analysis.

Gene symbol	GenBank ID	Forward primer	Reverse primer	Product size (bp)
*Il6*	NM_012589	AGCGATGATGCACTGTCAGA	GGAACTCCAGAAGACCAGAGC	127
*Il1b*	NM_031512	GACTTCACCATGGAACCCGT	GGAGACTGCCCATTCTCGAC	104
*Tnf*	NM_012675	GAACTCAGCGAGGACACCAA	GCCAGTGTATGAGAGGGACG	124

**Notes.**

Il6 interleukin 6 Il1b interleukin 1b Tnf tumor necrosis factor

### Immunohistochemistry for IL-6 and TNF-α in the intestine

Intestinal sections were fixed in 10% neutral buffered formalin, cast in paraffin, sliced into 4 µm sections and placed onto microscope slides. After deparaffinization, slides were immersed in PBS. Then, sections were stained with anti-IL-6 (sc-57315, 1:100, Santa Cruz Biotechnology, Dallas, TX) and anti-TNF-α (sc-52746, 1:100, Santa Cruz Biotechnology, Dallas, TX) at 4 °C overnight. Slides were then incubated with horseradish peroxidase (HRP)-conjugated secondary antibody (1:2000, Santa Cruz Biotechnology, Dallas, TX) for 1 h at room temperature. Immunolabeling was visualized with 0.05% diaminobenzidine (DAB). A Nikon 80i microscope (Nikon) was used to capture the images, and Nikon Elements (Nikon) software was used for image processing. Three slides were analyzed for each rat, and eight rats were included in each group.

### Statistical analysis

Data are shown as the mean ±SD. Statistical analyses were calculated with Student’s *t*-test for the difference between two groups and with one-way ANOVA followed by Tukey’s post hoc test for the difference among groups. GraphPad Prism 6 (GraphPad Software Inc., CA, USA) was used for data analysis. *P* < 0.05 was defined as significant.

## Results

### The effect of sitagliptin intervention on food intake, energy intake, body weight and blood glucose in pregnancy and lactation on dams

**Figure 2:**
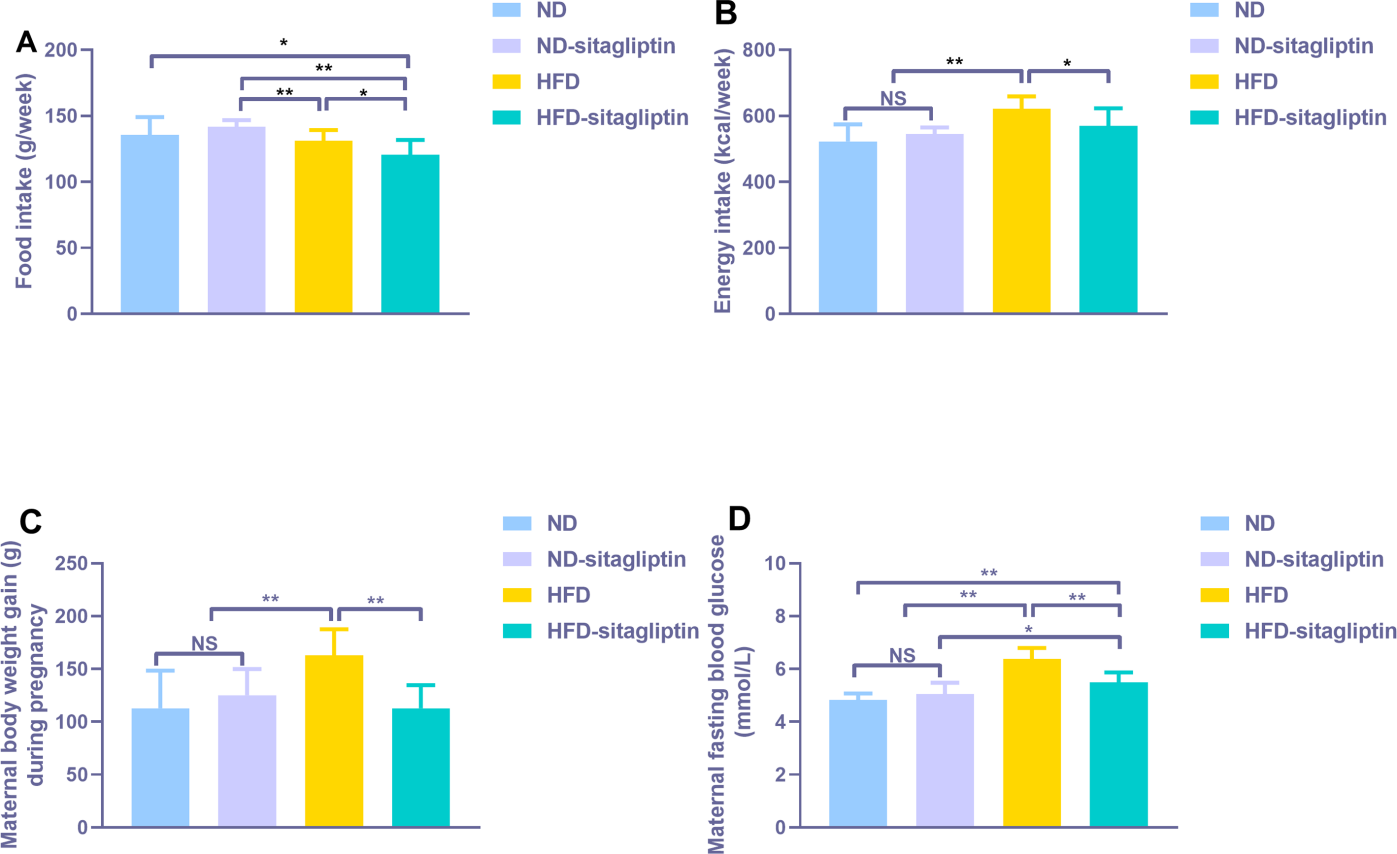
Effect of sitagliptin on maternal food intake (A), energy intake (B), body weight gain (C) and blood glucose (D). (A) Maternal food intake during pregnancy. (B) Maternal energy intake during pregnancy. (C) Maternal body weight gain during pregnancy. (D) Maternal fasting blood glucose at weaning time. Values are mean ± S.D. (*n* = 8). * *p* < 0.05, ** *p* < 0.01, ns not significant. ND: normal diet; HFD: high fat diet.

Food intake from HFD and HFD-sitagliptin reduced compared with ND-sitagliptin (*p* < 0.01, Fig. 2A) during pregnancy. However, energy intake of HFD dams was higher than that of the ND and ND-sitagliptin dams (*p* < 0.01, Fig. 2B). Accordingly, HFD dams gained significantly more weight than ND and ND-sitagliptin dams during pregnancy (*p* < 0.01, Fig. 2C). Sitagliptin intervention reduced dam food intake, energy intake and weight gain of HFD dams during pregnancy (*p* < 0.05*or*0.01, Figs. 2A, 2B, 2C). HFD dams had higher fasting blood glucose than ND and ND-sitagliptin dams at weaning time (*p* < 0.01, Fig. 2D); however, sitagliptin intervention reduced fasting blood glucose of HFD dams at weaning (*p* < 0.01, Fig. 2D).

### The effect of maternal sitagliptin intervention on metabolism in offspring

No difference in birth weight was observed among pups from ND, ND-sitagliptin, HFD, and HFD-sitagliptin dams (*p* > 0.05, Table 2). Body weight at 3 weeks of age was higher in both of male and female offspring from HFD dams than those from ND and ND-sitagliptin dams (*p* < 0.05 or 0.01, Table 2). Notably, maternal sitagliptin intervention reduced offspring body weight at 3 weeks of age (*p* < 0.01, Table 2). Only male pups from HFD dams had higher fasting blood glucose, blood glucose after oral glucose load, and area under the curve (AUC) of blood glucose than those from ND and ND-sitagliptin dams (*p* < 0.01, Table 2). Maternal sitagliptin intervention reduced fasting blood glucose, blood glucose after oral glucose load and AUC of blood glucose in male offspring from HFD dams (*p* < 0.01, Table 2). Additionally, the serum fasting insulin concentration and HOMA-IR index in pups from HFD dams were higher than those in male pups from ND and ND-sitagliptin dams (*p* < 0.01, Table 2), and maternal sitagliptin intervention reduced serum fasting insulin levels and the HOMA-IR index (*p* < 0.01, Table 2). There was no significant difference in fasting blood glucose, AUC of blood glucose, serum insulin and HOMA-IR among ND, ND-sitagliptin and HFD groups in female offspring (*p* > 0.05, Table 2). Female offspring in HFD-sitagliptin group had a slight increase than those from ND group in fasting blood glucose, AUC of blood glucose and HOMA-IR (*p* < 0.05, Table 2).

**Table 2:** Biochemical parameters of male and female offspring.

Biochemical parameters	Offspring gender	ND	ND-sitagliptin	HFD	HFD-sitagliptin
birth weight (g)	Male	6.85 ± 0.51	6.71 ± 0.54	7.08 ± 0.43	7.20 ± 0.58
	Female	6.70 ± 0.28	6.88 ± 0.34	6.96 ± 0.15	6.99 ± 0.40
body weight at weaning time (g)	Male	51.25 ± 4.53	53.75 ± 6.07	60.13 ± 4.16^∗∗#^	51.38 ± 4.21^&&^
	Female	48.13 ± 5.19	50.13 ± 4.70	61.50 ± 3.20^∗∗##^	48.50 ± 2.82^&&^
Fasting blood glucose (mmol/L) at weaning time	Male	4.94 ± 0.40	5.10 ± 0.69	6.23 ± 0.60^∗∗##^	5.03 ± 0.45^&&^
	Female	4.54 ± 0.53	4.70 ± 0.32	4.81 ± 0.20	5.00 ± 0.42^∗^
AUC (mmol/L/h)	Male	12.58 ± 0.67	12.98 ± 1.07	18.78 ± 0.63^∗∗##^	14.98 ± 0.98^∗∗##&&^
	Female	13.25 ± 0.81	14.11 ± 1.32	14.64 ± 1.78	15.05 ± 2.10^∗^
Insulin (ng/mL)	Male	0.66 ± 0.15	0.57 ± 0.08	1.26 ± 0.10^∗∗##^	0.71 ± 0.08^##&&^
	Female	0.51 ± 0.07	0.52 ± 0.11	0.56 ± 0.10	0.60 ± 0.07
HOMA-IR	Male	3.05 ± 0.74	2.75 ± 0.65	7.41 ± 1.13^∗∗##^	3.40 ± 0.48^##&&^
	Female	2.31 ± 0.46	2.35 ± 0.60	2.53 ± 0.60	2.83 ± 0.37^∗^

**Notes.**

Values are mean ± S.D. (*n* = 8). *p < 0.05, **p < 0.01 vs ND group, #*p* < 0.05, ##*p* < 0.01 vs ND-sitagliptin group, &&*p* < 0.01 vs HFD group. ND, normal diet; HFD, high fat diet.

### The effect of maternal early sitagliptin intervention on gene expression in male offspring intestine from gene array results and pathways

**Figure 3:**
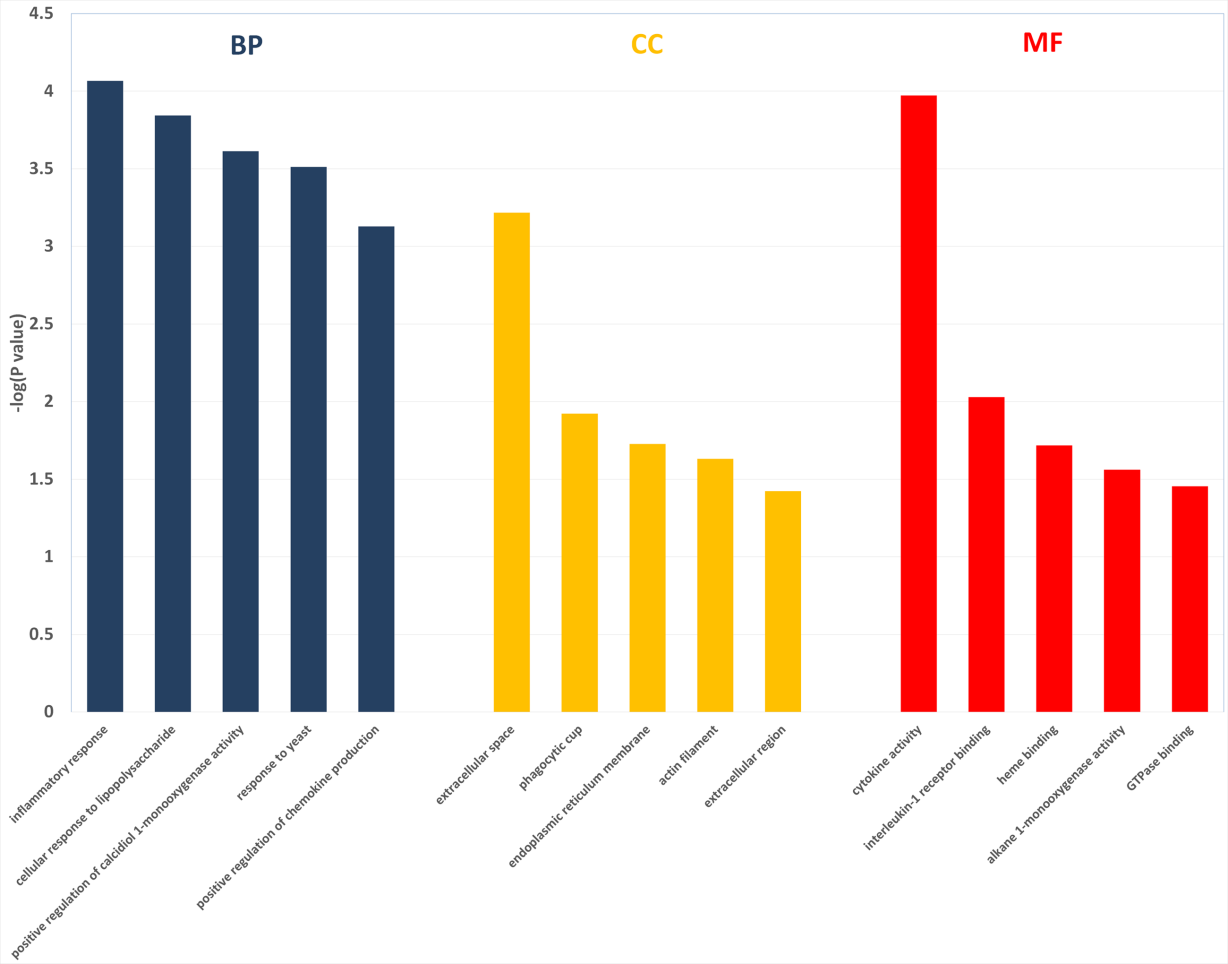
The enriched GO terms with differentially expressed genes between HFD group and HFD-sitagliptin group. Top five terms in biological process (BP), cellular component (CC) and molecular function (MP).

**Figure 4:**
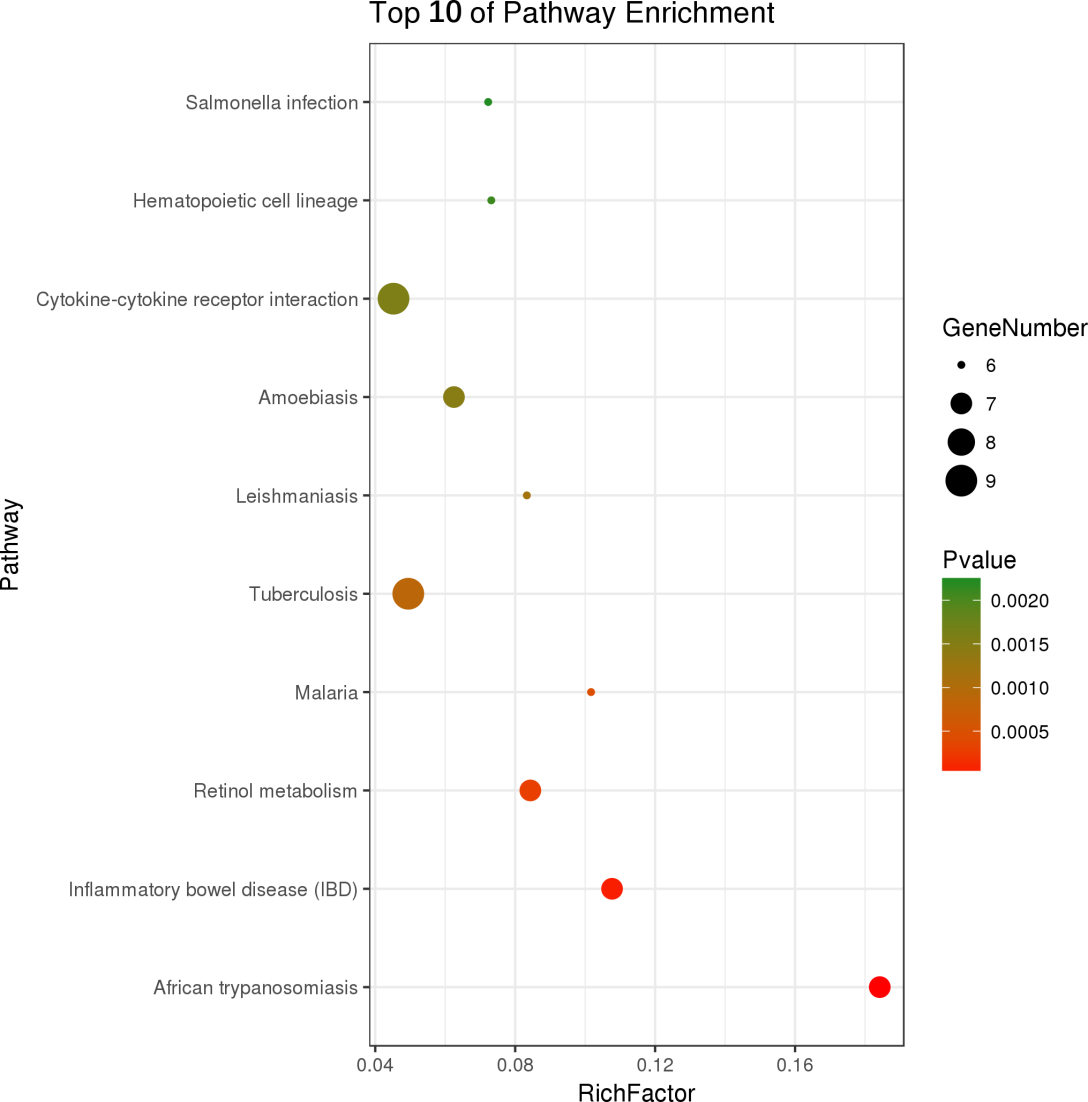
Top ten KEGG pathways enrichment point diagram in HFD-sitagliptin group compared with HFD group. The vertical axis represents the pathway name, the horizontal axis represents the Rich factor, the size of the dot indicates the number of genes expressed in the pathway, and the color of the dot corresponds to the different *p* value.

**Figure 5:**
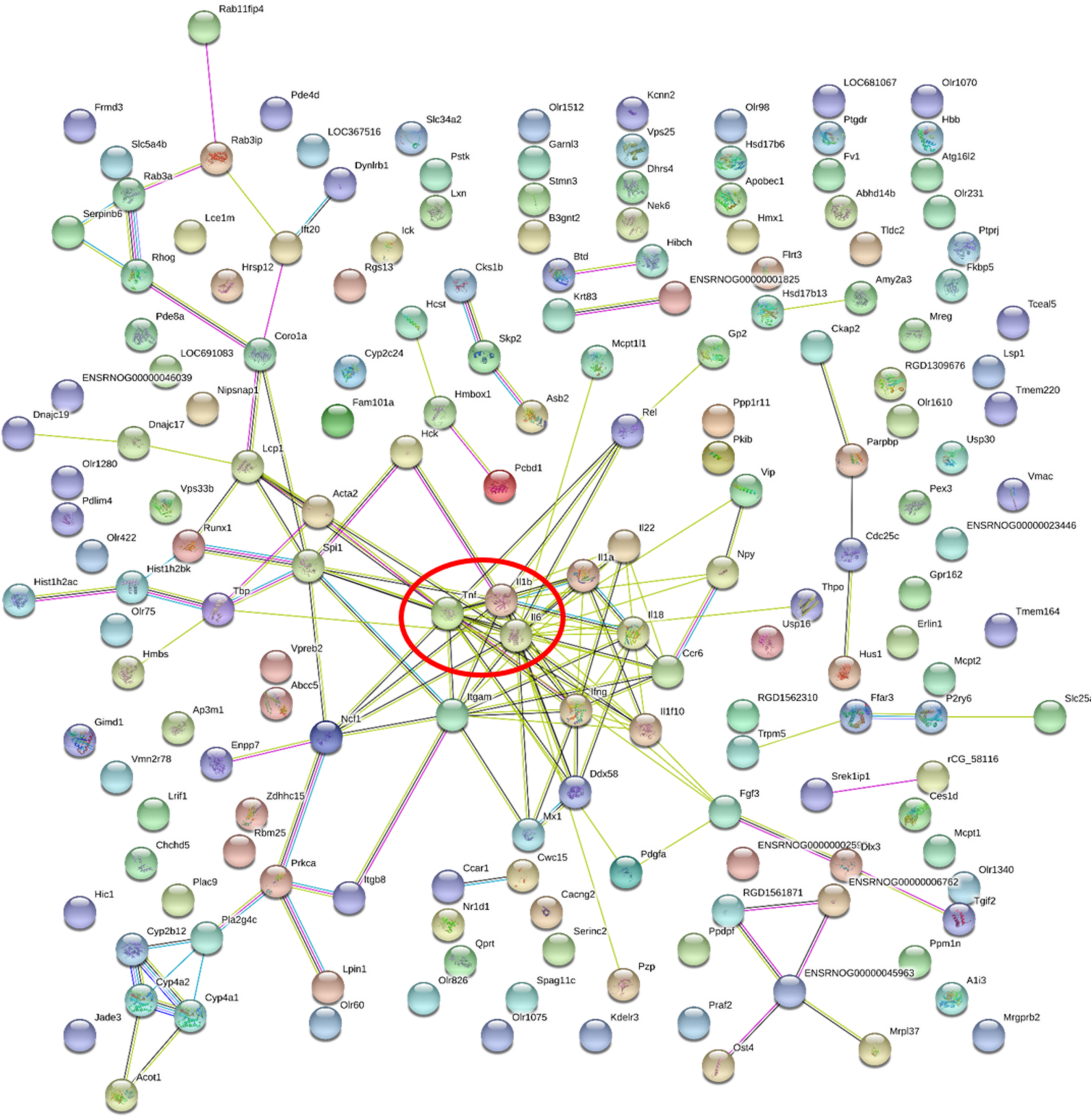
Gene-gene interaction network in HFD-sitagliptin group compared with HFD group. The nods stand for differentially expressed genes in HFD-sitagliptin group compared with HFD group. The lines stand for the interactions between two proteins.

Two hundred and one genes were differentially expressed in the intestines of pups from HFD-sitagliptin dams compared with those of pups from HFD dams (>1.50-fold, *p* < 0.05; 119 upregulated and 82 downregulated). To investigate the possible regulatory mechanisms of genes affected by maternal early sitagliptin intervention on offspring intestine, we analyzed biological processes, molecular functions, and cellular components through Gene Ontology and enriched KEGG pathways by DAVID. The results showed that differentially expressed genes between the HFD and HFD-sitagliptin groups were enriched in the following biological processes: inflammatory response, cellular response to lipopolysaccharide, positive regulation of calcidiol 1-monooxygenase activity, response to yeast, and positive regulation of chemokine production (*p* < 0.0001, Fig. 3, Table 3). They are mainly enriched in the following molecular functions: cytokine activity, interleukin-1 receptor binding, heme binding, alkane 1-monooxygenase activity, and GTPase binding (*p* < 0.05, Fig. 3, Table 3). Significant cellular components were extracellular space, phagocytic cup, endoplasmic reticulum membrane, actin filament, and extracellular region (*p* < 0.05, Fig. 3, Table 3). Enriched KEGG pathways by DAVID displayed in the KEGG pathway database showed that genes affected by maternal sitagliptin intervention on offspring intestine were significantly enriched in African trypanosomiasis, inflammatory bowel disease, retinol metabolism, malaria, tuberculosis, leishmaniasis, amoebiasis, cytokine-cytokine receptor interaction, hematopoietic cell lineage, and salmonella infection (*p* < 0.005, Fig. 4, Table 4). In STRING analysis, all differentially expressed genes were mapped in one network. In this network figure, *Il1b*, *Il6*, and *Tnf* were in the center of the differentially expressed gene network (Fig. 5, Table 5).

**Table 3:** The enriched GO terms with differentially expressed genes in HFD-sitagliptin group vs HFD group (*p* < 0.05).

Term ID	Term name	Count	*p* value	Fold enrichment	Catalog
GO:0006954	inflammatory response	12	8.59 × 10^−5^	4.42	biological processes
GO:0071222	cellular response to lipopolysaccharide	9	1.44 × 10^−4^	5.90	biological processes
GO:0060559	positive regulation of calcidiol 1-monooxygenase activity	3	2.44 × 10^−4^	109.59	biological processes
GO:0001878	response to yeast	4	3.07 × 10^−4^	29.22	biological processes
GO:0032722	positive regulation of chemokine production	4	7.45 × 10^−4^	21.91	biological processes
GO:0005615	extracellular space	24	6.08 × 10^−4^	2.16	cellular components
GO:0001891	phagocytic cup	3	1.19 × 10^−2^	17.80	cellular components
GO:0005789	endoplasmic reticulum membrane	11	1.87 × 10^−2^	2.34	cellular components
GO:0005884	actin filament	4	2.33 × 10^−2^	6.50	cellular components
GO:0005576	extracellular region	12	3.77 × 10^−2^	1.99	cellular components
GO:0005125	cytokine activity	9	1.07 × 10^−4^	6.15	Molecular function
GO:0005149	interleukin-1 receptor binding	3	9.36 × 10^−3^	20.14	Molecular function
GO:0020037	heme binding	6	1.91 × 10^−2^	3.88	Molecular function
GO:0018685	alkane 1-monooxygenase activity	2	2.74 × 10^−2^	71.61	Molecular function
GO:0051020	GTPase binding	3	3.51 × 10^−2^	10.07	Molecular function

**Table 4:** The enriched KEGG pathway with differentially expressed genes in HFD-sitagliptin group vs HFD group (*p* < 0.005).

Pathway ID	Pathway term	Count	Fold enrichment	*p* value	Involved genes
rno05143	African trypanosomiasis	7	16.40	3.25 × 10^−6^	PRKCA, IL6, TNF, IL18, IFNG, IL1B, HBB
rno05321	Inflammatory bowel disease (IBD)	7	9.59	7.67 × 10^−5^	IL6, TNF, IL18, IFNG, IL1B, IL22, IL1A
rno00830	Retinol metabolism	7	7.51	2.99 × 10^−4^	CYP4A2, CYP4A1, DHRS4, LOC100362350, HSD17B6, CYP2C24, CYP2B12
rno05144	Malaria	6	9.05	4.68 × 10^−4^	IL6, TNF, IL18, IFNG, IL1B, HBB
rno05152	Tuberculosis	9	4.40	8.83 × 10^−4^	LSP1, CORO1A, IL6, TNF, IL18, IFNG, IL1B, IL1A, ITGAM
rno05140	Leishmaniasis	6	7.42	1.16 × 10^−3^	TNF, NCF1, IFNG, IL1B, IL1A, ITGAM
rno05146	Amoebiasis	7	5.56	1.46 × 10^−3^	PRKCA, IL6, TNF, SERPINB6, IFNG, IL1B, ITGAM
rno04060	Cytokine-cytokine receptor interaction	9	4.02	1.56 × 10^−3^	IL6, TNF, CCR6, IL18, IFNG, IL1B, IL22, IL1A, THPO
rno04640	Hematopoietic cell lineage	6	6.51	2.09 × 10^−3^	IL6, TNF, IL1B, IL1A, ITGAM, THPO
rno05132	Salmonella infection	6	6.43	2.20 × 10^−3^	IL6, IL18, IFNG, IL1B, IL1A, RHOG

**Table 5:** A list of genes with connective degree no less than ten in the STRING network.

Gene accession	Gene symbol	Gene name	Degree
NM_012589.2	*Il6*	interleukin 6	22
NM_012675.3	*Tnf*	tumor necrosis factor	17
NM_031512.2	*Il1b*	interleukin 1 beta	13
NM_012711.1	*Itgam*	integrin subunit alpha M	12
NM_138880.2	*Ifng*	interferon gamma 1	11
NM_019165.1	*Il18*	interleukin 18	10
NM_001005892.2	*Spi1*	Spi-1 proto-oncogene	10

### The effect of maternal sitagliptin intervention on gene expression in male offspring intestine from real-time PCR

**Figure 6:**
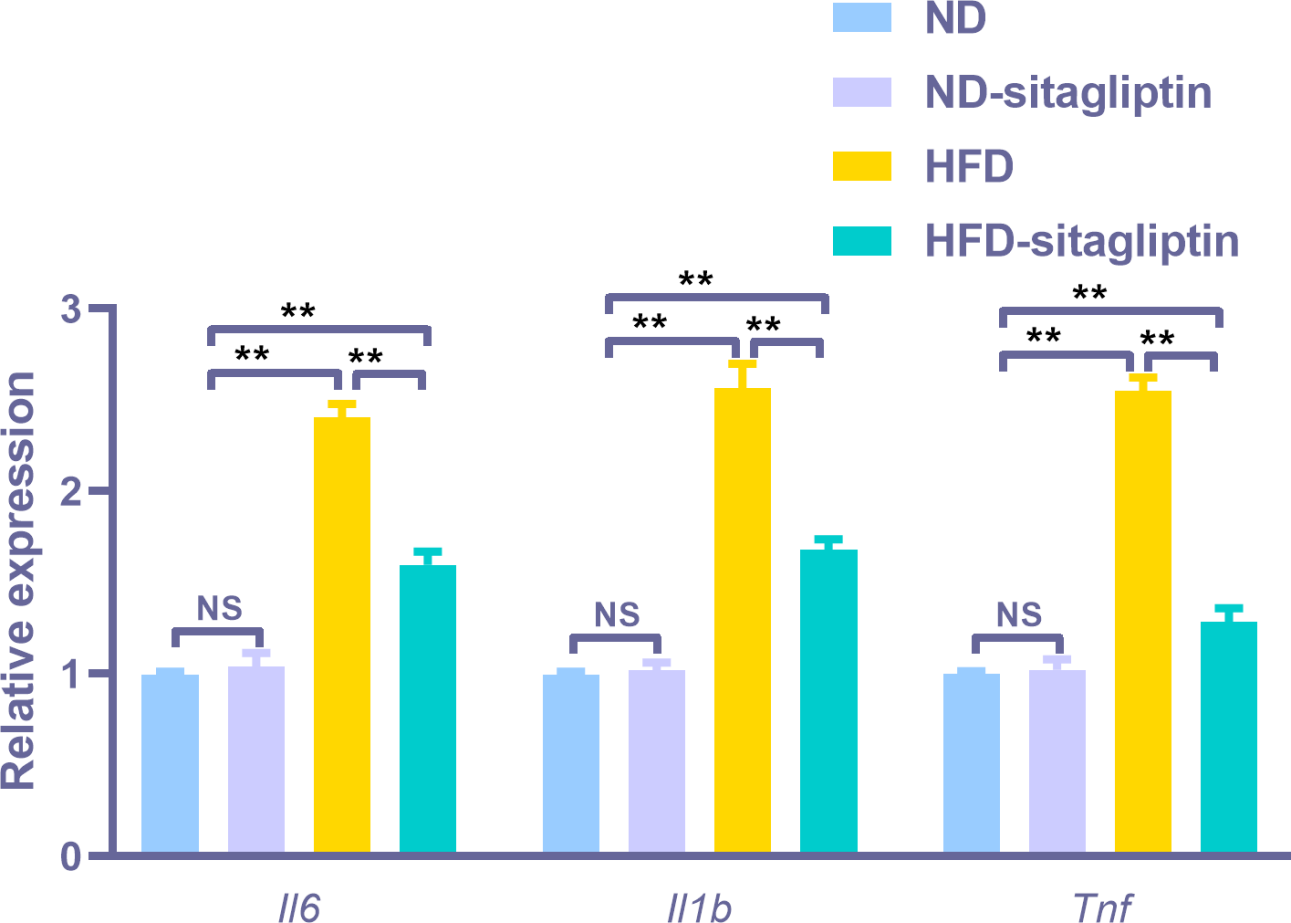
Confirmation of three representative differentially expressed genes (*Il6*, *Il1b*, *Tnf*) by qPCR. Values are mean ± S.D. (*n* = 8). ** *p* < 0.01, ^ns^ not significant. ND: normal diet; HFD: high fat diet.

To assess the reliability of the array hybridization results, three differentially expressed genes were quantified using real-time PCR. Gene array GO analysis showed that the inflammatory response was among the top biological processes. Moreover, cytokine-cytokine receptor interactions were among the top ten KEGG pathways. Proinflammatory markers *Il6*, *Il1b* and *Tnf* were in the center of the differentially expressed gene network. Thus, we selected these cytokines to validate the gene array results. *Il6*, *Il1b*, and *Tnf* expression levels were increased in the intestines of male offspring from HFD dams compared to those from ND and ND-sitagliptin dams (*p* < 0.01, Fig. 6). Interestingly, maternal sitagliptin intervention reduced *Il6*, *Il1b*, and *Tnf* expression levels in male offspring intestines form HFD dams (*p* < 0.01, Fig. 6).

### The effect of maternal sitagliptin intervention on proinflammatory markers IL-6 and TNF-α protein expression in male offspring intestines by immunohistochemical staining

**Figure 7:**
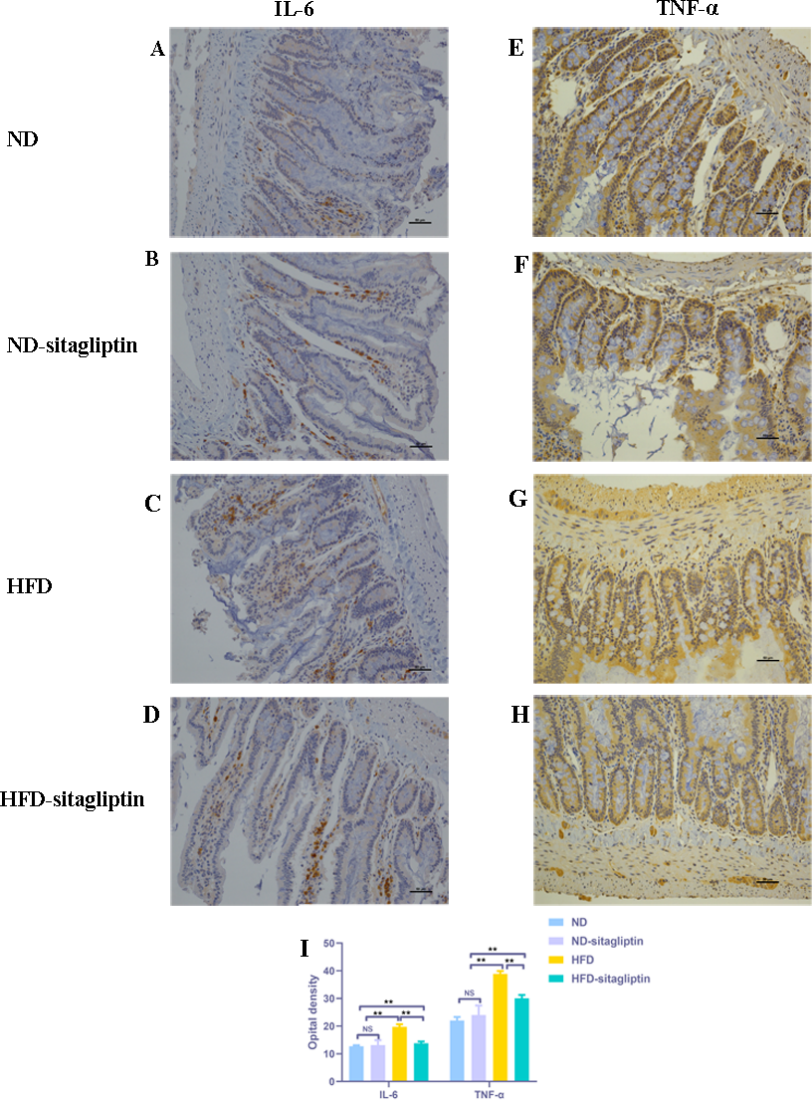
Effect of maternal sitagliptin on intestinal IL-6 and TNF-α expression in male offspring. (A–D) Immunostaining for IL-6 (200X) in ND, ND-sitagliptin, HFD, and HFD-sitagliptin group, (E–H) Immunostaining for TNF-α (200X) in ND, ND-sitagliptin, HFD, and HFD-sitagliptin group, (I) Optical density of IL-6 and TNF-α in intestine. Values are mean ± S.D. (*n* = 8). ** *p* < 0.01, ^ns^ not significant. ND: normal diet; HFD: high fat diet.

Consistent with the findings by real-time PCR, IL-6 and TNF-α expression in male offspring intestines from HFD dams was increased, compared with those from ND and ND-sitagliptin dams (*p* < 0.01, Fig. 7). However, maternal sitagliptin intervention reduced the immunoreactivity of IL-6 and TNF-α in male offspring from HFD dams (*p* < 0.01, Fig. 7).

### The effect of maternal sitagliptin intervention on serum proinflammatory cytokines IL-6, TNF-α and anti-proinflammatory cytokine IL-10 in male offspring

**Figure 8:**
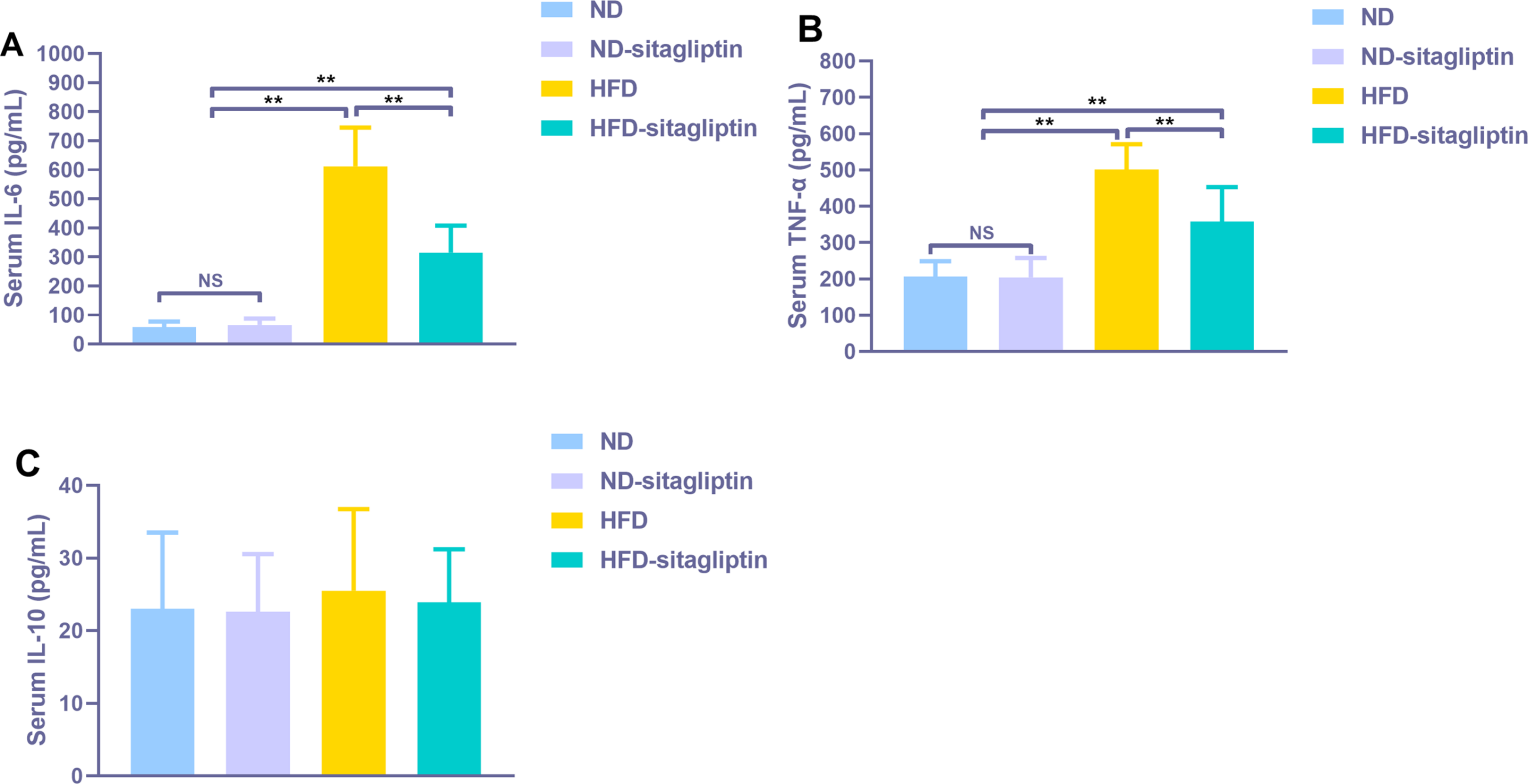
Effect of maternal sitagliptin on serum pro-inflammatory cytokines IL-6, TNF-α and anti-inflammotory cytokine IL-10 in male offspring. (A) Serum IL-6, (B) Serum TNF-α, (C) Serum IL-10. Values are mean ± S.D. (*n* = 8). **p < 0.01, ^ns^not significant. ND: normal diet; HFD: high fat diet.

Serum proinflammatory cytokines IL-6 and TNF-α increased significantly in male rat offspring from HFD dams (*p* < 0.01, Fig. 8), Maternal sitagliptin treatment reduced serum IL-6 and TNF-α levels in male offspring from HFD dams (*p* < 0.01, Fig. 8). However, there was no difference in serum anti-proinflammatory cytokine IL-10 levels among four groups (*p* > 0.05, Fig. 8).

## Discussion

Our results showed that sitagliptin had no significant effect on body weight and glucose metabolism of mother rats with normal diet and their pups. HFD increased energy intake and body weight gain during pregnancy, whereas sitagliptin intervention significantly reduced energy intake and body weight gain during pregnancy. A previous study also provided evidence that preconception sitagliptin treatment reduced body weight gain during pregnancy in a HFD-induced obese rodent model (Dennison, Eslinger & Reimer, 2017). In addition, we found that sitagliptin intervention improved maternal glucose homeostasis. One pilot study evaluated sitagliptin-metformin combined therapy in glucose-impaired women with a history of gestational diabetes mellitus (GDM) (Elkind-Hirsch et al., 2018). Sitagliptin-metformin is superior to metformin alone in improving glycemia in this prediabetic gestational female population (Elkind-Hirsch et al., 2018). Another clinical trial administered sitagliptin to Chinese GDM patients in their 2nd trimester (Sun et al., 2017). Sixteen weeks of sitagliptin administration significantly lowered fasting plasma glucose levels (Sun et al., 2017).

In addition, our findings revealed that a maternal HFD increased male and female offspring body weight at weaning. Consistent with our findings, other researchers also found that maternal high-fat diet exposure did not change birth weight but increased weaning weight in both female and male offspring (Ribaroff et al., 2017). However, our result showed that only male offspring from HFD dams had higher fasting blood glucose, glucose intolerance and insulin resistance. Previous paper also indicated the impact of offspring sex on blood glucose and insulin level is conflicting (Aldhous et al., 2015; Zheng et al., 2016). The potential mechanism lies in different responses to maternal diet, such as hormone release (Aiken & Ozanne, 2013). Next, we evaluated the effect of maternal sitagliptin intervention on offspring glucose metabolism. We found that maternal sitagliptin intervention attenuated glucose metabolism and insulin resistance in male offspring at weaning. However, intervening before pregnancy with sitagliptin did not have a significant effect on glucose metabolism (Dennison, Eslinger & Reimer, 2017). These different results may be due to differences in sitagliptin treatment time and duration.

Increasing evidence has revealed that maternal diet significantly affects infant microbiota composition (Nash, Frank & Friedman, 2017). A maternal HFD reduced the relative composition of *Campylobacter*, *Helicobacter*, and *Bacteroidetes* (Ma et al., 2014) and increased *Lachnospiraceae* and *Clostridiales* (Myles et al., 2013) in offspring. Importantly, changing to a control diet cannot completely reverse the key bacterial composition in pups (Ma et al., 2014). Bacterial composition disorders may affect biological processes in the gut. Currently, the gut is considered an important organ for whole body immune/metabolic health. Our results are the first to evaluate the effects of maternal sitagliptin intervention on offspring intestines. We observed that maternal sitagliptin intervention increased the expression of 119 genes and reduced the expression of 82 genes in male offspring intestine. Abundant studies have revealed that exposure to a high-fat diet causes significant alterations in intestinal gene expression (De Wit et al., 2011; Desmarchelier et al., 2012; Steegenga et al., 2012; Tremblay et al., 2013; Steegenga et al., 2017) found that maternal exposure to a Western-style (WS) diet during the perinatal period altered intestinal gene expression. These differentially expressed genes play an important role in intestinal development and functioning.

Interestingly, our findings show that maternal sitagliptin intervention mainly affected the inflammatory response biological process and cytokine-cytokine receptor interaction pathway. We observed dysregulated *Il6*, *Il1b* and *Tnf* gene expression in the intestines of male offspring in the HFD group at weaning, and remarkably, intestines of male offspring in the HFD-sitagliptin group exhibited reduced *Il6*, *Il1b*, and *Tnf* gene expression. Correspondingly, immunohistochemistry results also showed that maternal sitagliptin intervention reversed increased IL-6 and TNF-α levels in male offspring intestines affected by a maternal HFD. More interesingly, maternal sitagliptin intervention reduced serum IL-6 and TNF-α in male rat offspring. However, maternal sitagliptin did not affect offspring serum IL-10 levels. T2DM has been considered a disease condition with a low-grade inflammation state (Wellen & Hotamisligil, 2005). Glucose metabolism and food intake content are related to inflammatory status (Greco et al., 2014; Silveira et al., 2018), such as hypoglycemia and high fat diet (Arya et al., 2010). Proinflammatory pattern of upregulated IL-6 was observed in adipose tissue from GDM women (Lorenzo-Almoros et al., 2019; Wolf et al., 2004). In an animal model, a maternal high-fat diet not only induces cytokines in maternal serum and placenta involving IL-1β, TNF-α and MCP-1 (Ashino et al., 2012; Frias et al., 2011) but also enhances the level of TNF-α in adipocytes (Murabayashi et al., 2013) and increases IL-6 and TNF-α levels in the liver (Bruce et al., 2009; Oben et al., 2010). Adipose derived cytokines such as IL-6 and TNF-α elevated in obese adolescents and children (Syrenicz et al., 2006). The accumulation of activated macrophages within adipose tissue enlarged adipocytes of obese animals and humans (Weisberg et al., 2003; Xu et al., 2003). IL-6 and TNF-α may interact with each other. TNF-α functions at adipose perhaps stimulate the secretion of IL-6 (Kern et al., 2001). We found the reduced body weight in sitagliptin treated mother rats and their offspring. Thus, sitagliptin may inhibit low-grade chronic inflammation through reducing adipose tissue mass. In addition to the liver and adipose tissue (Hotamisligil, 2006), the gut may show dysbiosis and a certain degree of inflammation involved with gut barrier function disorder and hyperpermeability (Slyepchenko et al., 2016). For instance, gut-derived inflammation is involved with intestinal lipopolysaccharide (LPS) hyperpermeability (Lucas & Maes, 2013). Some evidence shows that a HFD increases proinflammatory cytokine expression in the intestine (Ding et al., 2010; Kim et al., 2012). Maternal HFD increased offspring serum IL-6 and TNF-α levels, even switch to normal diet after weaning in offspring (Kačarević et al., 2017; Peric Kacarevic et al., 2016). This indicates that inflammatory response was provoked in offspring by prenatal HFD. And HFD also elevated the concentration of LPS which can induce insulin resistance (Cani et al., 2007). Antibiotic treatment can lower inflammatory markers, decrease glucose intolerance and caecal LPS levels in obese mice (Cani et al., 2008). Moreover, HFD-induced inflammatory markers are directly transferred to the offspring via dam circulation or alter the quality and quantity of milk production during lactation (Bautista et al., 2016; Saben et al., 2014). Therefore, maternal sitagliptin intervention may inhibit proinflammatory cytokines IL-6 and TNF-α expression in the intestine to moderate intestinal permeability to LPS, leading to attenuate glucose dysmetabolism in offspring.

There is still some limitations of this study need to be stated. Nutrient composition of milk in dams during lactation may also play a role in offspring health. The milk of obese rats contained more energy than that from lean rats (Rolls et al., 1986). The effect of maternal HFD during lactation on milk composition is conflicting, because of different milk collection time-point. Butruille et al. reported that maternal HFD during lactation had no effect on rat global breast milk composition (Butruille et al., 2019). However, others found that milk from HFD dams had higher fat content at postnatal day (PND) 10 and 21(Purcell et al., 2011). And pups from HFD dams consumed more than pups from normal control diet dams (Purcell et al., 2011). For technical limitation, we did not test breast milk composition, consumption and neonatal rat blood glucose. Thus, we cannot eliminate the effect of maternal sitaglition on breast milk composition and consumption.

## Conclusions

In conclusion, we demonstrated that maternal sitagliptin intervention provides protection to offspring glucose metabolism and that improvement of intestinal proinflammatory cytokines IL-6 and TNF-α expression may be involved in the mechanism. The intestinal proinflammatory cytokines IL-6 and TNF-α pathway may be a novel target for reversing offspring glucose abnormalities affected by *in utero* overnutrition.

##  Supplemental Information

10.7717/peerj.10310/supp-1 Supplemental Information 1 Raw Data

10.7717/peerj.10310/supp-2 Supplemental Information 2 MIAME checklist

## Abbreviations

AUC: area under the curve
AIN: America Institute of Nutrition
DAVID: Database for Annotation, Visualization, and integrated Discovery
DPP: dipeptidyl peptidase
FDA: Food and Drug Administration
GLP: glucagon-like peptide
GO: Gene Ontology
HDF: high-fat diet
HOMA-IR: homeostasis model assessment of insulin resistance
IL-6: interleukin-6
IR: insulin resistance
KEGG: Kyoto Encyclopedia of Genes and Genomes
ND: normal diet
OGTT: oral glucose tolerance test
SD: Sprague-Dawley
T2DM: type 2 diabetes
Tnf-α: tumor necrosis factor alpha

## Additional Information and Declarations

### Microarray Data Deposition

The following information was supplied regarding the deposition of microarray data: The data is available at NCBI Gene Expression Omnibus (GEO): GSE134070.

### Data Availability

The following information was supplied regarding data availability: The raw measurements are available in the Supplemental File.

## References

[ref-1] Aiken CE, Ozanne SE (2013). Sex differences in developmental programming models. Reproduction.

[ref-2] Aldhous MC, Reynolds RM, Campbell A, Linksted P, Lindsay RS, Smith BH, Seckl JR, Porteous DJ, Norman JE (2015). Sex-differences in the metabolic health of offspring of parents with diabetes: a record-linkage study. PLOS ONE.

[ref-3] Arya F, Egger S, Colquhoun D, Sullivan D, Pal S, Egger G (2010). Differences in postprandial inflammatory responses to a ‘modern’ v. traditional meat meal: a preliminary study. British Journal of Nutrition.

[ref-4] Ashino NG, Saito KN, Souza FD, Nakutz FS, Roman EA, Velloso LA, Torsoni AS, Torsoni MA (2012). Maternal high-fat feeding through pregnancy and lactation predisposes mouse offspring to molecular insulin resistance and fatty liver. Journal of Nutritional Biochemistry.

[ref-5] Bautista CJ, Montano S, Ramirez V, Morales A, Nathanielsz PW, Bobadilla NA, Zambrano E (2016). Changes in milk composition in obese rats consuming a high-fat diet. British Journal of Nutrition.

[ref-6] Bergman A, Ebel D, Liu F, Stone J, Wang A, Zeng W, Chen L, Dilzer S, Lasseter K, Herman G, Wagner J, Krishna R (2007). Absolute bioavailability of sitagliptin, an oral dipeptidyl peptidase-4 inhibitor, in healthy volunteers. Biopharmaceutics and Drug Disposition.

[ref-7] Bianco-Miotto T, Craig JM, Gasser YP, Van Dijk SJ, Ozanne SE (2017). Epigenetics and DOHaD: from basics to birth and beyond. Journal of Developmental Origins of Health and Disease.

[ref-8] Boucher BJ, Leung PS (2015). Maternal high-fat-diet programs rat offspring liver fatty acid metabolism: might reduced vitamin D availability due to increases in maternal body fat contribute to this effect?. Lipids.

[ref-9] Bruce KD, Cagampang FR, Argenton M, Zhang J, Ethirajan PL, Burdge GC, Bateman AC, Clough GF, Poston L, Hanson MA, McConnell JM, Byrne CD (2009). Maternal high-fat feeding primes steatohepatitis in adult mice offspring, involving mitochondrial dysfunction and altered lipogenesis gene expression. Hepatology.

[ref-10] Butruille L, Marousez L, Pourpe C, Oger F, Lecoutre S, Catheline D, Görs S, Metges CC, Guinez C, Laborie C, Deruelle P, Eeckhoute J, Breton C, Legrand P, Lesage J, Eberlé D (2019). Maternal high-fat diet during suckling programs visceral adiposity and epigenetic regulation of adipose tissue stearoyl-CoA desaturase-1 in offspring. International Journal of Obesity.

[ref-11] Cani PD, Bibiloni R, Knauf C, Waget A, Neyrinck AM, Delzenne NM, Burcelin R (2008). Changes in gut microbiota control metabolic endotoxemia-induced inflammation in high-fat diet-induced obesity and diabetes in mice. Diabetes.

[ref-12] Cani PD, Neyrinck AM, Fava F, Knauf C, Burcelin RG, Tuohy KM, Gibson GR, Delzenne NM (2007). Selective increases of bifidobacteria in gut microflora improve high-fat-diet-induced diabetes in mice through a mechanism associated with endotoxaemia. Diabetologia.

[ref-13] De Wit NJ, Boekschoten MV, Bachmair EM, Hooiveld GJ, De Groot PJ, Rubio-Aliaga I, Daniel H, Muller M (2011). Dose-dependent effects of dietary fat on development of obesity in relation to intestinal differential gene expression in C57BL/6J mice. PLOS ONE.

[ref-14] Deacon CF, Holst JJ (2013). Dipeptidyl peptidase-4 inhibitors for the treatment of type 2 diabetes: comparison, efficacy and safety. Expert Opinion on Pharmacotherapy.

[ref-15] Dennison CA, Eslinger AJ, Reimer RA (2017). Preconception prebiotic and sitagliptin treatment in obese rats affects pregnancy outcomes and offspring microbiota, adiposity, and glycemia. Frontiers in Endocrinology.

[ref-16] Desmarchelier C, Dahlhoff C, Keller S, Sailer M, Jahreis G, Daniel H (2012). C57Bl/6 N mice on a western diet display reduced intestinal and hepatic cholesterol levels despite a plasma hypercholesterolemia. BMC Genomics.

[ref-17] Ding S, Chi MM, Scull BP, Rigby R, Schwerbrock NM, Magness S, Jobin C, Lund PK (2010). High-fat diet: bacteria interactions promote intestinal inflammation which precedes and correlates with obesity and insulin resistance in mouse. PLOS ONE.

[ref-18] Elkind-Hirsch KE, Paterson MS, Shaler D, Gutowski HC (2018). Short-term sitagliptin-metformin therapy is more effective than metformin or placebo in prior gestational diabetic women with impaired glucose regulation. Endocrine Practice.

[ref-19] Eriksson JG, Sandboge S, Salonen MK, Kajantie E, Osmond C (2014). Long-term consequences of maternal overweight in pregnancy on offspring later health: findings from the Helsinki Birth Cohort Study. Annals of Medicine.

[ref-20] Frias AE, Morgan TK, Evans AE, Rasanen J, Oh KY, Thornburg KL, Grove KL (2011). Maternal high-fat diet disturbs uteroplacental hemodynamics and increases the frequency of stillbirth in a nonhuman primate model of excess nutrition. Endocrinology.

[ref-21] Gallwitz B (2007). Sitagliptin: profile of a novel DPP-4 inhibitor for the treatment of type 2 diabetes. Drugs Today.

[ref-22] Greco M, Chiefari E, Montalcini T, Accattato F, Costanzo FS, Pujia A, Foti D, Brunetti A, Gulletta E (2014). Early effects of a hypocaloric, Mediterranean diet on laboratory parameters in obese individuals. Mediators of Inflammation.

[ref-23] Hotamisligil GS (2006). Inflammation and metabolic disorders. Nature.

[ref-25] Jornayvaz FR, Vollenweider P, Bochud M, Mooser V, Waeber G, Marques-Vidal P (2016). Low birth weight leads to obesity, diabetes and increased leptin levels in adults: the CoLaus study. Cardiovascular Diabetology.

[ref-26] Kačarević ŽP, Grgić A, Šnajder D, Bijelić N, Belovari T, Cvijanović O, Blažičević V, Radić R (2017). Different combinations of maternal and postnatal diet are reflected in changes of hepatic parenchyma and hepatic TNF-alpha expression in male rat offspring. Acta Histochemica.

[ref-27] Kern PA, Ranganathan S, Li C, Wood L, Ranganathan G (2001). Adipose tissue tumor necrosis factor and interleukin-6 expression in human obesity and insulin resistance. American Journal of Physiology, Endocrinology and Metabolism.

[ref-28] Kieffer TJ, McIntosh CH, Pederson RA (1995). Degradation of glucose-dependent insulinotropic polypeptide and truncated glucagon-like peptide 1 in vitro and in vivo by dipeptidyl peptidase IV. Endocrinology.

[ref-29] Kim KA, Gu W, Lee IA, Joh EH, Kim DH (2012). High fat diet-induced gut microbiota exacerbates inflammation and obesity in mice via the TLR4 signaling pathway. PLOS ONE.

[ref-30] Kleinert M, Clemmensen C, Hofmann SM, Moore MC, Renner S, Woods SC, Huypens P, Beckers J, De Angelis MH, Schürmann A, Bakhti M, Klingenspor M, Heiman M, Cherrington AD, Ristow M, Lickert H, Wolf E, Havel PJ, Müller TD, Tschöp MH (2018). Animal models of obesity and diabetes mellitus. Nature Reviews Endocrinology.

[ref-31] Lorenzo-Almoros A, Hang T, Peiro C, Soriano-Guillen L, Egido J, Tunon J, Lorenzo O (2019). Predictive and diagnostic biomarkers for gestational diabetes and its associated metabolic and cardiovascular diseases. Cardiovasc Diabetol.

[ref-32] Lovshin JA, Drucker DJ (2009). Incretin-based therapies for type 2 diabetes mellitus. Nature Reviews Endocrinology.

[ref-33] Lucas K, Maes M (2013). Role of the toll like receptor (TLR) radical cycle in chronic inflammation: possible treatments targeting the TLR4 pathway. Molecular Neurobiology.

[ref-34] Ma J, Prince AL, Bader D, Hu M, Ganu R, Baquero K, Blundell P, Alan Harris R, Frias AE, Grove KL, Aagaard KM (2014). High-fat maternal diet during pregnancy persistently alters the offspring microbiome in a primate model. Nature Communications.

[ref-35] Mega C, De Lemos ET, Vala H, Fernandes R, Oliveira J, Mascarenhas-Melo F, Teixeira F, Reis F (2011). Diabetic nephropathy amelioration by a low-dose sitagliptin in an animal model of type 2 diabetes (Zucker diabetic fatty rat). Experimental Diabetes Research.

[ref-36] Murabayashi N, Sugiyama T, Zhang L, Kamimoto Y, Umekawa T, Ma N, Sagawa N (2013). Maternal high-fat diets cause insulin resistance through inflammatory changes in fetal adipose tissue. European Journal of Obstetrics & Gynecology and Reproductive Biology.

[ref-37] Myles IA, Fontecilla NM, Janelsins BM, Vithayathil PJ, Segre JA, Datta SK (2013). Parental dietary fat intake alters offspring microbiome and immunity. Journal of Immunology.

[ref-38] Nair AB, Jacob S (2016). A simple practice guide for dose conversion between animals and human. Journal of Basic and Clinical Pharmacy.

[ref-39] Nash MJ, Frank DN, Friedman JE (2017). Early Microbes modify immune system development and metabolic homeostasis—the restaurant hypothesis revisited. Frontiers in Endocrinology.

[ref-40] Oben JA, Mouralidarane A, Samuelsson AM, Matthews PJ, Morgan ML, McKee C, Soeda J, Fernandez-Twinn DS, Martin-Gronert MS, Ozanne SE, Sigala B, Novelli M, Poston L, Taylor PD (2010). Maternal obesity during pregnancy and lactation programs the development of offspring non-alcoholic fatty liver disease in mice. Journal of Hepatology.

[ref-41] Ohta T, Toriniwa Y, Ryumon N, Inaba N, Hirao T, Yamanaka S, Maeno T, Sakakibara W, Sumikawa M, Chiba K, Nakamura A, Miyajima K, Fatchiyah F, Yamada T (2017). Maternal high-fat diet promotes onset of diabetes in rat offspring. Journal of Animal Science.

[ref-42] Peric Kacarevic Z, Snajder D, Maric A, Bijelic N, Cvijanovic O, Domitrovic R, Radic R (2016). High-fat diet induced changes in lumbar vertebra of the male rat offsprings. Acta Histochemica.

[ref-43] Pratley RE, Salsali A (2007). Inhibition of DPP-4: a new therapeutic approach for the treatment of type 2 diabetes. Current Medical Research and Opinion.

[ref-44] Purcell RH, Sun B, Pass LL, Power ML, Moran TH, Tamashiro KL (2011). Maternal stress and high-fat diet effect on maternal behavior, milk composition, and pup ingestive behavior. Physiology and Behavior.

[ref-45] Reimann F, Habib AM, Tolhurst G, Parker HE, Rogers GJ, Gribble FM (2008). Glucose sensing in L cells: a primary cell study. Cell Metabolism.

[ref-46] Ribaroff GA, Wastnedge E, Drake AJ, Sharpe RM, Chambers TJG (2017). Animal models of maternal high fat diet exposure and effects on metabolism in offspring: a meta-regression analysis. Obesity Reviews.

[ref-47] Rolls BA, Gurr MI, Van Duijvenvoorde PM, Rolls BJ, Rowe EA (1986). Lactation in lean and obese rats: effect of cafeteria feeding and of dietary obesity on milk composition. Physiology and Behavior.

[ref-48] Saben JL, Bales ES, Jackman MR, Orlicky D, MacLean PS, McManaman JL (2014). Maternal obesity reduces milk lipid production in lactating mice by inhibiting acetyl-CoA carboxylase and impairing fatty acid synthesis. PLOS ONE.

[ref-49] Samaha MM, Said E, Salem HA (2019). A comparative study of the role of crocin and sitagliptin in attenuation of STZ-induced diabetes mellitus and the associated inflammatory and apoptotic changes in pancreatic beta-islets. Environmental Toxicology and Pharmacology.

[ref-50] Silveira BKS, Oliveira TMS, Andrade PA, Hermsdorff HHM, Rosa COB, Franceschini S (2018). Dietary pattern and macronutrients profile on the variation of inflammatory biomarkers: scientific update. Cardiology Research and Practice.

[ref-51] Slyepchenko A, Maes M, Machado-Vieira R, Anderson G, Solmi M, Sanz Y, Berk M, Kohler CA, Carvalho AF (2016). Intestinal dysbiosis, gut hyperpermeability and bacterial translocation: missing links between depression, obesity and type 2 diabetes. Current Pharmaceutical Design.

[ref-52] Steegenga WT, De Wit NJ, Boekschoten MV, Ijssennagger N, Lute C, Keshtkar S, Bromhaar MM, Kampman E, De Groot LC, Muller M (2012). Structural, functional and molecular analysis of the effects of aging in the small intestine and colon of C57BL/6J mice. BMC Medical Genomics.

[ref-53] Steegenga WT, Mischke M, Lute C, Boekschoten MV, Lendvai A, Pruis MG, Verkade HJ, Van de Heijning BJ, Boekhorst J, Timmerman HM, Plosch T, Muller M, Hooiveld GJ (2017). Maternal exposure to a Western-style diet causes differences in intestinal microbiota composition and gene expression of suckling mouse pups. Molecular Nutrition & Food Research.

[ref-54] Sun X, Zhang Z, Ning H, Sun H, Ji X (2017). Sitagliptin down-regulates retinol-binding protein 4 and reduces insulin resistance in gestational diabetes mellitus: a randomized and double-blind trial. Metabolic Brain Disease.

[ref-55] Syrenicz A, Garanty-Bogacka B, Syrenicz M, Gebala A, Walczak M (2006). Low-grade systemic inflammation and the risk of type 2 diabetes in obese children and adolescents. Neuroendocrinology Letters.

[ref-56] Tremblay AJ, Lamarche B, Guay V, Charest A, Lemelin V, Couture P (2013). Short-term, high-fat diet increases the expression of key intestinal genes involved in lipoprotein metabolism in healthy men. American Journal of Clinical Nutrition.

[ref-57] Virally M, Blickle JF, Girard J, Halimi S, Simon D, Guillausseau PJ (2007). Type 2 diabetes mellitus: epidemiology, pathophysiology, unmet needs and therapeutical perspectives. Diabete et Metabolisme.

[ref-58] Weisberg SP, McCann D, Desai M, Rosenbaum M, Leibel RL, Ferrante Jr AW (2003). Obesity is associated with macrophage accumulation in adipose tissue. Journal of Clinical Investigation.

[ref-59] Wellen KE, Hotamisligil GS (2005). Inflammation, stress, and diabetes. Journal of Clinical Investigation.

[ref-60] Williams R, Karuranga S, Malanda B, Saeedi P, Basit A, Besancon S, Bommer C, Esteghamati A, Ogurtsova K, Zhang P, Collagiuri S (2020). Global and regional estimates and projections of diabetes-related health expenditure: results from the International Diabetes Federation Diabetes Atlas, 9(th) edition. Diabetes Research and Clinical Practice.

[ref-61] Wojcicka G, Zareba M, Warpas A, Jamroz-Wisniewska A, Rusek M, Czechowska G, Beltowski J (2019). The effect of exenatide (a GLP-1 analog) and sitagliptin (a DPP-4 inhibitor) on plasma platelet-activating factor acetylhydrolase (PAF-AH) activity and concentration in normal and fructose-fed rats. European Journal of Pharmacology.

[ref-62] Wolf M, Sauk J, Shah A, Vossen Smirnakis K, Jimenez-Kimble R, Ecker JL, Thadhani R (2004). Inflammation and glucose intolerance: a prospective study of gestational diabetes mellitus. Diabetes Care.

[ref-63] Xu H, Barnes GT, Yang Q, Tan G, Yang D, Chou CJ, Sole J, Nichols A, Ross JS, Tartaglia LA, Chen H (2003). Chronic inflammation in fat plays a crucial role in the development of obesity-related insulin resistance. Journal of Clinical Investigation.

[ref-64] Yokomizo H, Inoguchi T, Sonoda N, Sakaki Y, Maeda Y, Inoue T, Hirata E, Takei R, Ikeda N, Fujii M, Fukuda K, Sasaki H, Takayanagi R (2014). Maternal high-fat diet induces insulin resistance and deterioration of pancreatic beta-cell function in adult offspring with sex differences in mice. American Journal of Physiology, Endocrinology and Metabolism.

[ref-65] Zhang Q, Sun X, Xiao X, Zheng J, Li M, Yu M, Ping F, Wang Z, Qi C, Wang T, Wang X (2018). Maternal chromium restriction induces insulin resistance in adult mice offspring through miRNA. International Journal of Molecular Medicine.

[ref-66] Zheng J, Xiao X, Zhang Q, Yu M, Xu J, Wang Z (2014). Maternal high-fat diet modulates hepatic glucose, lipid homeostasis and gene expression in the PPAR pathway in the early life of offspring. International Journal of Molecular Sciences.

[ref-67] Zheng J, Zhang Q, Mul JD, Yu M, Xu J, Qi C, Wang T, Xiao X (2016). Maternal high-calorie diet is associated with altered hepatic microRNA expression and impaired metabolic health in offspring at weaning age. Endocrine.

[ref-68] Zhou X, Wang W, Wang C, Zheng C, Xu X, Ni X, Hu S, Cai B, Sun L, Shi K, Chen B, Zhou M, Chen G (2019). DPP4 inhibitor attenuates severe acute pancreatitis-associated intestinal inflammation via Nrf2 signaling. Oxidative Medicine and Cellular Longevity.

