# Supplementary material for: Maternal sitagliptin treatment attenuates offspring glucose metabolism and intestinal proinflammatory cytokines IL-6 and TNF-α expression in male rats
Source: PeerJ. 2020 Nov 11;8:e10310. doi: 10.7717/peerj.10310 (PMC7666563; doi:10.7717/peerj.10310)
Supplement: Supplemental Information 2 [file peerj-08-10310-s002.docx]

**Miame Checklist**

**Experiment Design**

Female Sprague-Dawley (SD) rats were fed a normal diet (ND) or a high-fat diet (HFD) for 4 weeks before mating. Dams fed an ND were divided into two subgroups: the ND only group and ND-sitagliptin group (n = 8 for each group). Meanwhile, HFD pregnant rats were randomized to one of two groups: HFD group (HFD alone) and the HFD-sitagliptin group (HFD combined with 10 mg/kg/day sitagliptin treatment) during pregnancy and lactation. Glucose metabolism was assessed in offspring at weaning. Male offspring intestinal gene expression levels were investigated using microarray.

**Raw data availability**

Raw data of six microarray experiments were submitted to the NCBI Gene Expression Omnibus (GEO) database (accession number GSE134070). The platform is GPL17117

**Sample used**

The intestine of six offspring rats were used to perform microarray expermients.

**Measurement Data and Specifications**

Total RNA was isolated from the intestines of pups in the HFD and HFD-sitagliptin groups by using TRIzol reagent (Life Technologies Inc., Carlsbad, CA, USA). Gene expression in the intestine was detected by an Affymetrix GeneChip Rat Gene 2.0 ST whole transcript-based array (Affymetrix Technologies, Santa Clara, CA). Arrays were scanned using the GeneChip® Scanner 3000 Affymetrix). All arrays were uploaded into Genespring v12.5 (Agilent Tchnologies, USA) for data normalization, quality control and first-pass analysis. Differentially expressed genes were then identified through fold change as well as P value calculated with t-test. The threshold set for differentially regulated genes was a fold change>1.5 and a P value<0.05.
